# Recent progress on polymeric probes for formaldehyde sensing: a comprehensive review

**DOI:** 10.1080/14686996.2024.2423597

**Published:** 2024-11-12

**Authors:** Subhadip Roy, Swagata Pan, Priyadarsi De

**Affiliations:** Polymer Research Centre and Centre for Advanced Functional Materials, Department of Chemical Sciences, Indian Institute of Science Education and Research Kolkata, Mohanpur, India

**Keywords:** Formaldehyde, polymeric probe, fluorescent probe, fluorometric sensing, colorimetric sensing, Sensors and actuators

## Abstract

Formaldehyde (FA) is a reactive toxic volatile organic compound (VOC), produced both exogenously from the environment and endogenously within most organisms, and poses significant health risks to humans at elevated concentrations. Consequently, the development of reliable and sensitive FA sensing technologies is crucial for environmental monitoring, industrial safety, and public health protection. This review will provide a concise overview of FA sensing methodologies, highlighting key principles, sensing mechanisms, and recent advancements. The main aim of this review article is to comprehensively discuss recent advancements in FA sensors utilizing small molecules, nanoparticles, organic materials, and polymers, along with their successful applications across various fields, with particular emphasis on *in situ* FA sensing using polymeric probes due to their advantages over small molecular probes. Additionally, it will discuss prospects for future design and research in this area. We anticipate that this article will aid in the development of next-generation polymeric FA sensing probed with improved physicochemical properties.

## IMPACT STATEMENT

The main objective of this review article is to provide a comprehensive overview of formaldehyde sensing, highlighting the advantages of polymer-based sensors over other types of sensors.

## GRAPHICAL ABSTRACT

**Figure:**
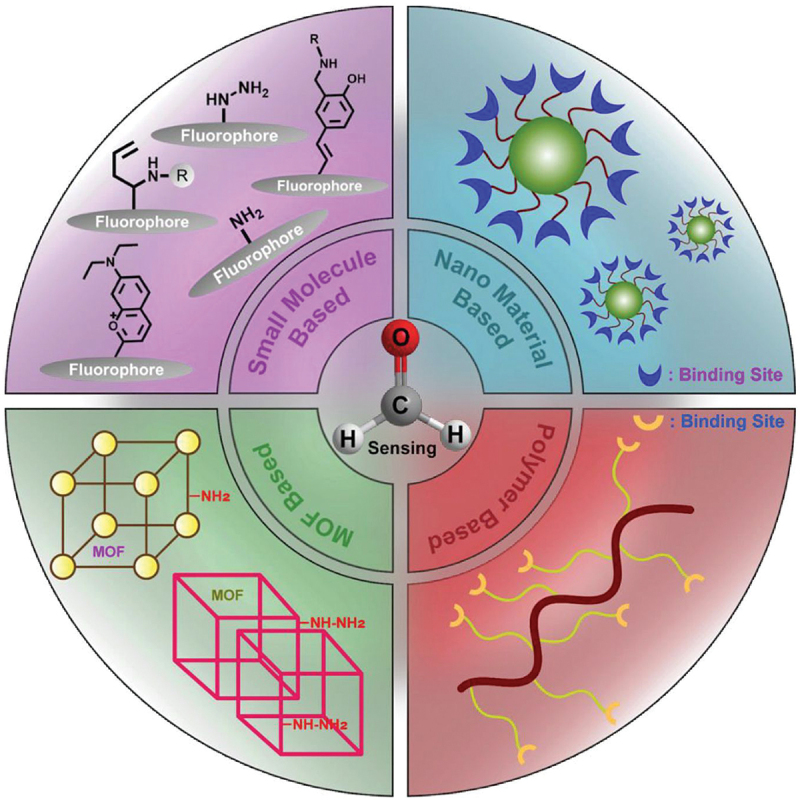

## 1. Introduction

Formaldehyde (FA) is the simplest and most reactive carbonyl species (RCS). Forty percent aqueous solution of FA is known as formalin. FA is extensively used in textiles, cosmetics, items of furniture, resins, preservatives, plastics, wood processing, industrial chemicals, etc., and is the chief anthropogenic source of FA [1]. Along with these, FA is also added to increase the shelf life of food items like fish, meat, fruits, and vegetables. Widespread applications of FA and formalin have made it one of the ubiquitous chemical contaminants in the environment, as FA is a known carcinogen that is extremely harmful to the environment and human health [2]. Significant demand for FA-based products in various applications made it the third largest indoor chemical pollutant mentioned by the World Health Organization (WHO).

In 2004, the International Agency for Research on Cancer (IARC) declared FA as one of the Grade-I carcinogenic [3]. According to the WHO, the maximum daily absorption of FA is limited up to 0.15 mg/kg of body weight, and the inhalation of FA is limited by restricting its concentration to 0.1 mg/m^3^ (0.08 ppm) in air [4]. 750 ppb is the standard permissible exposure limiting value listed by the Occupational Safety and Health Administration (OSHA), and the highest exposure value is considered 20 ppm for Immediately Dangerous to Life or Health (IDLH) [5,6]. Inhaling the gaseous FA released from FA-based products and taking foods with FA preservatives above the limits are the primary causes of exogenous FA in the human body.

FA is produced endogenously by the demethylation of macromolecules like deoxyribonucleic acid (DNA) and ribonucleic acid (RNA) [7]. Metabolism of sugars, lipids, and aldehydes in the sub-millimolar range *via* enzymatic pathways also produces FA, because a series of metabolic processes are catalyzed by oxidases and demethylases like semi-carbazide sensitive amine oxidase (SSAO) [8], and lysine-specific demethylase 1 (LSD1) [9]. Endogenous FA carries out key processes in cell homeostasis, including cell proliferation and memory formation. Nonetheless, under the influence of diverse external factors, an abnormal rise in FA concentration often leads to FA carbonyl stress resulting in degradation of neuronal networks by polymerization of *τ*-protein and hyperphosphorylation [10]. A small amount of FA causes headaches, sneezing, coughing, nausea, and throat irritation. In contrast, high concentration and long-term exposure to FA leads to severe injuries like allergic lung inflammation [11], hypertension [12], Alzheimer’s disease [13], and cancer [14]. Therefore, the development of a facile and sensitive method with a highly efficient probe having a fast response is required for detecting FA.

Given the concentration-dependent characteristics of FA and the key signalling pathway it regulates, the past decade has witnessed the development of technologies to precisely determine endogenous and exogenous FA levels. A variety of methods have been established to detect the mutagenic, teratogenic, and carcinogenic FA. The following detection methods are developed to spot FA: potentiometric [15], conductometry [16], sol-gel based [17], electrochemical biosensors [18], Raman spectroscopy [19], quartz crystal microbalance [20], gas chromatography [21], liquid chromatography [22], and transmission electron microscopy (TEM) [23]. The 2,4-dinitrophenylhydrazine coupled high-performance liquid chromatography (DNPH-HPLC) was also used for FA detection, especially in environmental and industrial settings [24]. This method produces stable derivatives, allowing for accurate quantification. Furthermore, the hydroxylamine detector tube method was introduced as a portable and easy-to-use for quick on-site detection [25]. However, due to the lack of sensitivity and precision needed for detailed analysis, these methods are better suited for routine analysis. Hydroxylamine/single-walled carbon nanotubes (SWCNT) based materials are also used as a chemiresistive FA gas sensor [26,27].

Luminescent imaging using spectroscopic techniques and fluorescent probes has attracted a lot of attention due to its facile operation, ease of use, low instrument cost, excellent penetration depth in tissues, high sensitivity, and convenience. An ideal probe must have the following features: (a) the presence of soluble or targeting groups, (b) colorimetric/fluorometric response, (c) a suitable reaction center, and (d) a reversible sensing reaction, such that the probe would be suitable for practical applications in redox biology (like organelle-specific and *in vivo* applications). Among all kinds of methods, recently developed fluorescent-based methods show better performance in terms of selectivity and sensitivity for FA detection in the solution phase [28]. In this regard, polymeric probes were developed for better fluorescence signal amplification with lower response time due to the presence of multiple recognition sites in a single polymer chain [29].

**Figure 1.**
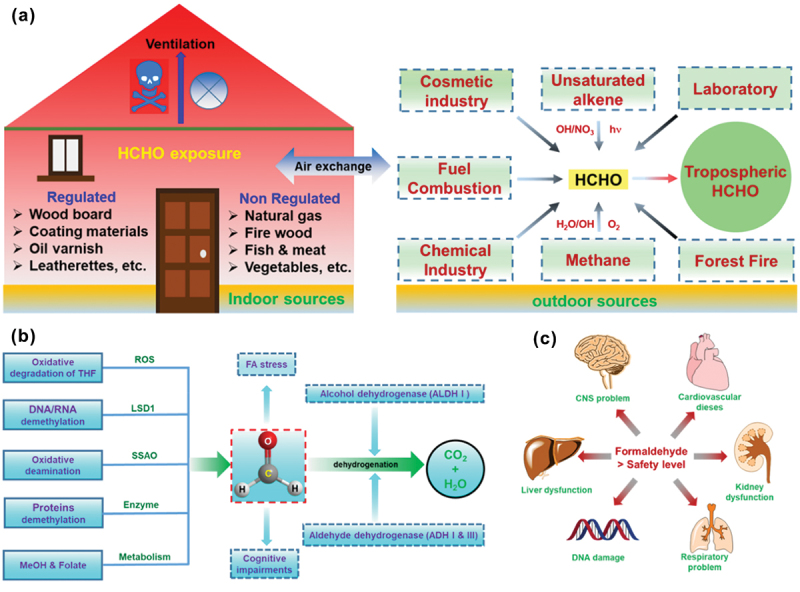
(a) Production of FA in nature (indoor and outdoor). (b) Formation and degradation of FA in the human body. (c) Harmful effects of FA.

A significant number of fluorescent probes have been reported for spotting FA. The reactive carbonyl species FA has superior electrophilicity and quickly participates in nucleophilic addition with thiol (−SH), hydroxyl (−OH), and amine (-NH_2_). Currently, chemo-sensors bearing the amine functional group as a binding site are extensively used to detect FA. The reported fluorescent sensors are primarily based on three types of reaction mechanisms; (i) 2-aza-Cope rearrangement reaction, followed by *β*-elimination [30], (ii) the reaction of aromatic amine to generate formimine [31], (iii) the reaction of aromatic hydrazine (-NHNH_2_) to form hydrazone (RCH=NR) [32,33]. The 2-aza-Cope rearrangement reaction transforms a homoallylic amine group into an aldehyde moiety after reacting with FA. The aromatic hydrazine reacts with FA to form aromatic hydrazine, whereas the amine functional group condenses with FA to produce formimine moiety. Small molecule-based FA sensors are often designed utilizing different response modes like turn-on/turn-off mechanisms, and ratiometric detection [34], along with including a variety of sensing mechanisms such as photoinduced electron transfer (PET) [35], intramolecular charge transfer (ICT) [36], spirocyclization [37], where the probe emits fluorescence signal only after reacting with FA. Some fluorescent sensors have limitations like: (i) low selectivity, (ii) low penetration depth in tissues, (iii) no specific targeting ability, (iv) long response time, and (v) high limit of detection (LOD). A low selectivity problem arises due to increasing interference from other carbonyl compounds due to nonspecific recognition. One-photon excitation is the major cause of low penetration depth in tissues, limiting the tracking of endogenous FA in deep regions of organs. No specific targeting ability appears because sensors without subcellular targeting moieties are unfavourable for detecting FA at the organelle level. Production of FA in nature, formation, and degradation of FA in the human body, and harmful effects of FA are summarized in Figure 1.

Recently, polymers have gained significant attention in the field of sensing to overcome the limitations in small molecule systems. Polymeric sensors offer a platform for simple functionalization and customization to include specific functional groups. This versatility allows researchers to design sensors that target specific molecules, such as formaldehyde, and adapt the sensor properties (e.g. sensitivity, selectivity) according to the desired application. In addition, solubility, biocompatibility, etc. can be tuned in polymer structures by suitable choice of monomer units or by post-polymerization functionalization [38]. Thus, water-soluble fluorescent polymeric probes with pendant primary amine groups are utilized as an alternative strategy for FA detection, eliminating the usage of hazardous and volatile organic solvents in detection investigations. Polymeric sensors are accessible for mass manufacturing due to the scalable and economically cheap nature of their synthesis and fabrication process. Polymeric materials can be created by many functional binding sites in the main backbone or side chain pendants [39]. Thus, polymeric probes can enhance their binding affinity and selectivity due to the presence of several recognition regions in the polymeric structure. The synergistic interactions between the low-concentration analyte and the large number of recognition binding sites rise through the polymeric network due to cooperative effects in polymeric probes [40]. The analyte interacts with the polymeric probe through numerous recognition sites at the same time, significantly increasing the total binding affinity compared to single-site probes [41]. Thus, polymeric sensors are suitable in terms of sensitivity, selectivity, stability, cost-effectiveness, and manufacturing control [42]. Our group reported polymeric probes for heavy metal sensing [43] and detection of toxic nitro-aromatic derivatives in an aqueous medium [44]. The desirable signal amplification and quick fluorescence response make polymeric probes extremely useful for different applications like metal sensing [45], diagnostics [46], biosensing [47], real-life monitoring, and detecting environmental or food pollutants [48]. This review aims to offer a thorough exploration of the chemistry involved in FA sensing, including detailed discussions on mechanisms and recent advancements from 2010 onward, highlighting significant breakthroughs in polymeric probes for FA sensing.

## 2. Detection of FA using small molecule-based sensors

### 2.1. FA sensing using 2-aza-Cope rearrangement

**Figure 2.**
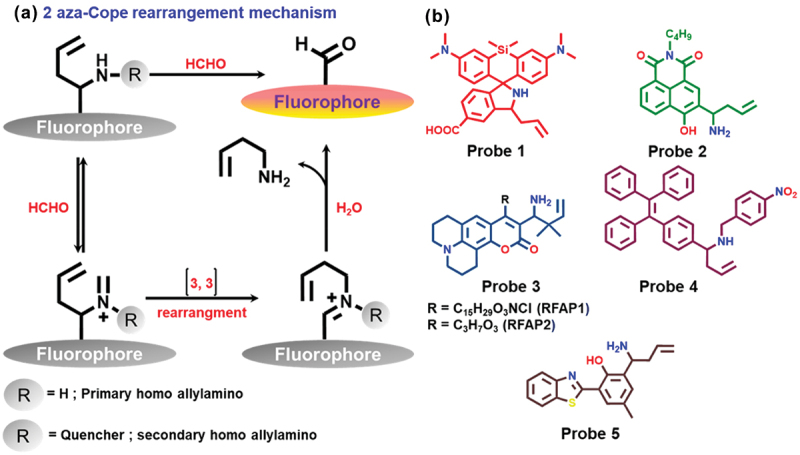
(a) Schematic representation of 2-aza-Cope rearrangement reaction. (b) Chemical structures of probes 1, 2, 3, 4, and 5.

The aza-Cope rearrangement reaction has been utilized as a successful method for detecting FA [49]. When an amine group (specifically a homoallylic) of the probe was reacted with FA, forming an iminium intermediate, which underwent a 2-aza-Cope sigmatropic rearrangement reaction and produced the desired fluorescent product upon hydrolysis (Figure 2a). Brewer and Chang reported a highly fluorescent probe **1** (Figure 2b) for FA sensing based on aza-Cope rearrangement mechanism [50]. A spiro-type silicon-rhodamine dye was designed for the detection of FA exogenously and endogenously. Probe **1** was in the closed-loop state of silicon-rhodamine dye and displayed weak fluorescence, but an 8-fold enhancement of fluorescence intensity in 30 min was observed after being treated with FA (at excitation of maximum wavelength 645 nm). This research group reported that probe **1** can detect different concentrations of exogenous FA in HEK293T cells. Moreover, probe **1** showed excellent selectivity for FA, good biocompatibility, and trans-membrane capacity. The *in vitro* detection limit for FA was calculated to be 5 μM.

Xie et al. reported a novel two-photon fluorescent probe **2** (Figure 2b) for sensing FA based on aza-Cope rearrangement [51]. They have chosen 4-hydroxyl 1,8-naphthalimide as a two-photon dye due to its high photostability and Stokes shifts. Around 30 nm blue shift was observed after the reaction with FA, in absorption as well as in emission spectrum. When the probe solution was added to the FA (5 mm), an approximately 20-fold fluorescence enhancement was observed after 3 h of incubation, and the detection limit was determined as 5 µM. Time-dependent density functional theory (TD-DFT) study supported the mechanism of ICT enhancement, during the reaction between probe **2** and FA. Moreover, probe **2** displayed a significant signal change when treated with FA *in vivo*.

Two coumarin-based probes (**RFAP-1** and **RFAP-2**) were designed for the selective detection FA in living systems [52]. The probes **RFAP-1** and **RFAP-2** (Figure 2b) enabled the ratiometric imaging of FA in biological systems. The excitation wavelength shifted 50 nm after incubation of 100 mm FA to the probe (**RFAP-1**). The *in vivo* detection limit was found to be 0.3 mm at a concentration of 10 mm probe. Both **RFAP-1** and **RFAP-2** displayed a variety of photophysical properties (high optical brightness) with visible excitation and emission spectrum. Specifically, the probe **RFAP-2** recognized the differences between wild-type cells and models with a gene knockout of ADH5 (FA-metabolizing enzyme).

Zhao et al. developed an aggregation-induced emission (AIE) based fluorescent probe **4** (Figure 2b) to detect gaseous FA [53]. The FA reactive moiety (homoallylic amine) and a fluorescence quencher moiety (4-nitrobenzyl) were embodied into tetraphenylethene (TPE) to develop probe **4**. The test plate was modeled by loading probe **4** directly on a high-performance thin-layer chromatography silica gel plate (HPTLC plate) to prepare a portable solid sensor for FA detection. The weak fluorescence of probe **4** was a result of PET between the electron donor (TPE) and the electron acceptor (4-nitrobenzyl) moieties. Probe **4** displayed a turn-on fluorescence response towards the gaseous FA, *via* 2-aza-Cope sigmatropic rearrangement. When gaseous FA was added to the test plate, they observed a colorimetric change under 365 nm UV light with a significant fluorescence (FL) intensity increase (∼8.7-fold, *λ*_em_ at 504 nm). The limit of detection for gaseous FA was found to be 0.036 mg/m^3^, which is lower than the air quality guideline value of gaseous FA. The test plate exhibited good selectivity in indoor air. FA test plates are safe, easily portable, and more convenient for detecting gaseous FA than other solution-based sensors.

In 2020, Hao *et al.* prepared a new excited-state intramolecular proton transfer-based probe **5** (benzothiazole-derivative) for FA sensing *via* 2-aza-Cope rearrangement mechanism (Figure 2b) [54]. Upon treatment with FA, the probe led to a significant red shift in both absorption (392 nm to 452 nm) and emission (492 nm to 552 nm) bands, and the color of the solution changed from colorless to yellowish in the naked eye *via* excited state intramolecular proton transfer (ESIPT). The detection limit of the probe was found to be 0.58 μM and showed a ~35.7 fold fluorescence intensity enhancement after reaction with FA. The probe specifically responded to FA over other reactive carbonyl species and relevant biological samples. Additionally, probe **5** was successfully used for ratiometric imaging of FA in zebrafish and living cells.

Recently, Tantipanjaporn et al. developed a ratiometric fluorescent probe based on ESIPT for rapid (within 10 min), selective, and sensitive detection of FA *via* the 2-aza-Cope rearrangement [55]. Liu et al. reported a cell-permeable fluorescent indicator that can detect FA in cancer cells using ‘multi-lock system key-and-lock’ strategy [56]. Li and coworkers prepared quinolizinium-based fluorescent probes for FA detection *via* a 2-aza-Cope rearrangement reaction. They demonstrated the application of these probes for detecting FA in mouse serum and utilized them in FA test strips [57]. Very recently, Tenney et al. developed an organelle-targetable, activity-based sensing platform for detecting endogenous FA in a breast cancer cell line [58]. Therefore, the search for FA sensors for different applications is still at the forefront.

### 2.2. Aromatic amine (Schiff)-based fluorescent probes

**Figure 3.**
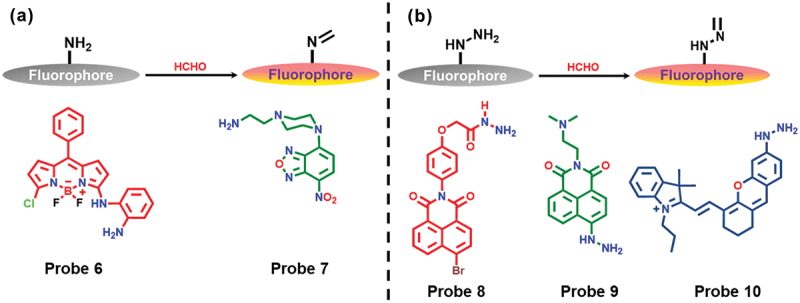
(a) Schematic representation of Schiff reaction and chemical structures of the probes 6, and 7. (b) Schematic representation of reaction between hydrazine and FA, and chemical structures of the probes 8, 9, and 10.

Schiff bases are formed by the condensation of an aldehyde or ketone with a primary amine. The chemical reaction between amine and FA leads to the formation of an imine bond, which shows an effect on emission and absorption properties. Recently, the development of aromatic amine-based fluorescent probes for FA sensing gained significant attention due to fast response time. In this section, we discuss the exploration of diverse amine derivatives anchored to fluorophores for the detection of FA (Figure 3a).

4,4-Difluoro-4-bora-3a,4a-diaza-*s*-indacene (BODIPY) based FA chemo-dosimeter probe **6** was developed by Cao et al. [59]. In the presence of FA, probe **6** induced a decrease in the absorption peak at 482 nm, accompanied by an increase in the peak at 525 nm. Upon the gradual addition of FA to the solution of probe **6**, a significant red shift in fluorescence was observed from 525 nm to 548 nm, along with a remarkable enhancement in fluorescence intensity. The authors conducted test kit experiments for detecting FA in environmental samples, revealing that probe **6** had a detection limit of 0.104 mm and exhibited high selectivity over other potentially interfering analytes. Notably, probe **6** was employed for monitoring and imaging FA in HeLa cells.

Gangopadhyay et al. synthesized a 2-aminoethyl piperazine conjugated 7-nitrobenz-2-oxa-1,3-diazole (NBD)-based small molecule (probe **7** in Figure 3a) for FA sensing in aqueous medium and gaseous phase [60]. The chemical reaction between probe **7** and FA in aqueous 4-(2-hydroxyethyl)-1-piperazineethanesulfonic acid (HEPES) buffer (pH 7.4) solution resulted in a color change (from orange to yellow) due to a decrease in absorbance value at 485 nm as well as a substantial fluorescence intensity enhancement was observed at 545 nm (51-fold). The probe **7** was very sensitive towards FA compared to other potential interferences (such as reactive carbonyl and oxygen species), except for glyoxal, which showed a slight response to probe **7**. The fluorescence intensity exhibited a linear relationship with FA concentrations ranging from 0 to 2.5 mm, with a low detection limit of 84 nM. Furthermore, probe **7** was utilized for detecting FA in plywood samples and for fluorescence imaging of FA in living HADF cells.

### 2.3. Methylenehydrazine-based sensors

The hydrazine group, possessing a nucleophilic free amino group, readily reacts with FA to produce methylenehydrazine due to the alpha effect. This structural conversion from free amino to methylenehydrazine inhibits the PET process from the free amino group to the fluorophore, resulting in spectral changes. Probes of this type are known for their rapid response to FA (Figure 3b). In 2018, Bi and coworkers prepared a simple probe **8** for FA sensing based on a fluorescence turn-on mechanism [61]. In the reaction between probe **8** and FA, the PET process gets quenched and the fluorescence signal turns on the green region due to the formation of the corresponding imine. Additionally, density functional theory (DFT) calculation supported the sensing mechanism where PET is off and fluorescence is on. The addition of 30 µM FA into probe **8** resulted in ~18.9-fold fluorescence enhancement at 525 nm. Probe **8** displayed fast detection of FA compared to other aldehydes and ketones, with a detection limit of 20 nM, and time was found to be 6 min. They also developed a paper strip-based method for FA detection. Upon the addition of FA to the probe **8** loaded paper, the color of the paper changed from blue to green under a 365 nm UV lamp. This probe was also able to image and detect FA in living cells.

A new turn-on fluorescent probe **9** (Figure 3b) was developed with a hydrazine group serving as a binding site for FA in living cells [62]. Upon the reaction of the hydrazine group with FA, probe **9** exhibited a substantial turn-on signal, emitting strong fluorescence. Probe **9** demonstrated high sensitivity and selectivity to FA in phosphate-buffered saline (PBS) solution. Additionally, it was capable of detecting both exogenous and endogenous FA in living cells. Utilizing probe **9**, they investigated the distribution of FA in cells and confirmed that endoplasmic reticulum stress could elevate the concentration of FA in living cells. They anticipated that probe **9** could serve as a valuable bioactive molecule tracking reagent for imaging FA, enabling further exploration of the physiological and pathological functions associated with FA.

In 2022, Ding et al. reported a near-infrared (NIR) fluorescent probe **10** (Figure 3b) to detect FA through the Schiff base reaction with hydrazine and FA *via* fluorescence off-on mechanism [63]. The photoluminescence performance of probe **10** in detecting FA was examined, revealing its high sensitivity and selectivity specifically for FA detection in solution. The probe displayed a rapid response towards FA within 10 min with a detection limit of 0.68 μM. Additionally, this hydrazine-based probe displayed better performance compared to the amine-based probe, in terms of reaction time and detection limit. Furthermore, probe **10** can be easily prepared as a paper chip for quick visualization of FA detection. Probe **10** also demonstrated excellent localization and tracing abilities for endogenous FA in zebrafish and mice, suggesting its potential as a diagnostic tool for diseases related to FA overexpression.

### 2.4. FA sensing via other reaction mechanisms

#### 2.4.1. Reversible fluorescent probe for SO_2_ and FA

**Figure 4.**
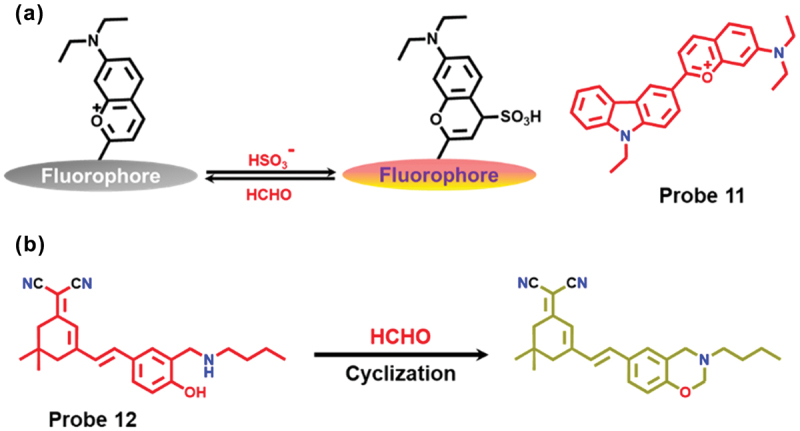
(a) Schematic representation of a reversible fluorescent probe for SO2 and FA detection (Probe 11). (b) FA detection using Probe 12 via cyclization mechanism and formation of benzoxazine derivatives.

Given the intimate and intricate relationship between sulfur dioxide (SO_2_) and FA in living systems, it becomes crucial to simultaneously monitor the dynamic concentrations of both [64]. A conventional probe was documented to concurrently detect SO_2_ alongside FA, where a carbazole-based fluorescent probe **11** was developed, capable of reversibly detecting SO_2_ and FA through the Michael-addition reaction and the interaction between nucleophilic bisulfite and electrophilic FA (Figure 4a) [65]. Probe **11** exhibited an exceptionally rapid response towards SO_2_ within 5 sec, with FA effectively restoring the response in less than 1 min. Moreover, the probe allowed for a reversible monitoring cycle of SO_2_ and FA, at least nine times *in vitro*. In addition, this robust molecular probe was applied to real-time tracking of endogenous SO_2_ and FA. It also facilitated reversible imaging of SO_2_ and FA in live zebrafish and mice. Thus, this research introduced a potent chemical tool for tracking the dynamic interaction of naturally occurring SO_2_ and FA.

#### 2.4.2. Detection of FA via cyclization reaction

A colorimetric and ratiometric fluorescent NIR-probe **12** was designed for the detection of FA based on the cyclization reaction [66,67]. Utilizing the synthetic approach of benzoxazine derivatives, the detection mechanism relied on a cyclization reaction for FA detection (Figure 4b). The probe exhibited a fast response, high sensitivity, and good selectivity to FA. The probe **12** was successfully utilized to image FA in the lysosomes of HeLa cells and zebrafish. The incorporation of a weakly basic morpholine group rendered the probe useful for detecting FA in cellular lysosomes.

Thus, representative FA-sensing probes have been discussed, which are based on aza-Cope, Schiff base, cyclization, and condensation reactions. A comparison of the photophysical properties is discussed before and after the reaction with FA in terms of reaction time, reactant concentration, pH, etc. From the above discussion, aza-Cope-based probes take longer sensing time compared to other organic small molecular probes. Table 1 summarizes different types of small molecule-based FA sensors with various properties and applications.

**Table 1.** Small molecule-based FA sensing probes.

Probe	Fluorophore	Sensing Mechanism	Photophysical Mechanism	Detection Limit	Time	Application	Ref.
1	Silicon rhodol julolidine-based	2-Aza-Cope	Turn-on/PET	5 µM	30 min	Cell imaging (Neuroscreen-1 cells and live HEK293TN cells)	50
2	Napthalimide	2-Aza-Cope	Turn-on/ICT	5 µM	-	Imaging zebrafish	51
3	Coumarin	2-Aza-Cope	Turn on	0.3 mm	-	Cell imaging	52
4	TPE	2-Aza-Cope	Turn-on/PET, AIE	0.036 mg/m^3^	60 min	gaseous FA detection	53
5	Benzothiazole	2-Aza-Cope	Turn-on/ ESIPT	0.58 μM	<3 min	Imaging zebrafish	54
6	BODIPY	Schiff base	Turn-on/ICT, ESIPT	0.104 mm	100 min	Cell imaging	59
7	NBD	Schiff base	Turn-on/PET	84 nM	-	Cell imaging	60
8	Naphthalimide	Hydrazine	Turn-on (two-photon)/PET	20 nM	6 min	Cell imaging	61
9	1,8-Naphthalimide	Hydrazine	Turn-on/PET	65 nM	-	Cell imaging (HeLa cells)	62
10	NIR-Dye	Hydrazine	Turn-on/PET	0.68 μM	10 min	Zebrafish and mice	63
11	Carbazole	Chalcone	Turn-on	-	<1 min	Zebrafish and mice	65
12	NIR-Dye	Cyclization	Turn-on/off	4.5 μM	<1 min	HeLa cells and zebrafish	67

## 3. Nanomaterial-based FA sensors

Luminescent nanomaterials have garnered recent interest for their potential applications in optical displays, bioimaging, security systems, and sensing. Nanomaterial-based sensors offer benefits, such as increased surface area and distinctive nanoscale characteristics. Few reports have been published on FA sensing utilizing different luminescent nanomaterials. Li and coworkers reported FA-responsive nitrogen-rich graphene quantum dots (NGQDs) encapsulated with *N*-rich organic species using a simple hydrothermal method [68]. Notably, these functionalized NGQDs exhibited a fascinating color oscillation in ambient air, and their fluorescence emission could be reversibly switched on and off through redox reactions. Additionally, by modifying the surface states of NGQDs, the photoluminescence (PL) emission transformed from excitation-independent to excitation-dependent. The limit of detection for FA was found to be 0.15 μM. Thus, the functionalized NGQDs with their unique optical and luminescent properties served as fluorescent sensors for FA detection and bioimaging purposes.

**Figure 5.**
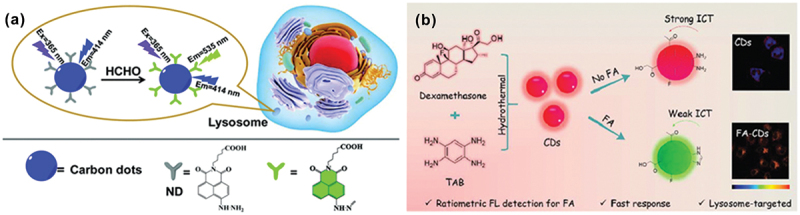
(a) Lysosome targeting ratiometric probe for FA sensing by CD-ND. Reprinted with permission from ref. [69]. Copyright (2019), Royal Society of Chemistry. (b) The schematic representation of selective and sensitive sensing of FA in lysosomes of living cells with fast response. Reprinted with permission from ref. [70]. Copyright (2020), Elsevier.

A ratiometric fluorescent nanoprobe based on naphthalimide (ND) functionalized carbon dots (CDs) was prepared for FA detection in lysosomes [69]. Carbon dots in a conventional hydrothermal method were developed by mixing methionine and citric acid in water. Next, the naphthalimide derivative was covalently linked with CDs and finally attached with hydrazine moiety to prepare the probe (CD-ND). TEM images showed a uniform distribution with spherical morphology of CDs and CD-ND. Interaction of FA with the probe gradually increased the emission intensity at 535 nm (~10-fold), and the limit of detection was found to be 0.34 μM. This probe was capable of tracking lysosomes and selectively detecting FA in HeLa cells (Figure 5a). A ‘synthesis-modification integration’ approach was used for preparing N and P co-doped CDs, using *N*-(phosphonomethyl)iminodiacetic acid (PMIDA) and branched polyethyleneimine (BPEI) as binary dopants [70]. In treatment with FA, the fluorescence intensity of the CDs increased with noticeable bathochromic shifts in both the fluorescence and absorption spectra. This response was attributed to the AIE ‘turn-on’ response of the CDs and the enhancement of *π*-conjugation, due to the formation of FA-induced Schiff base in this unique N and P co-doped electron-rich system. The detection limit was found to be 0.47 μM along with distinct color changes from colorless to light yellow. This dual-mode design concept was introduced as a simple, low-cost, and accurate route for constructing various CD sensors toward FA (Figure 5b).

**Figure 6.**
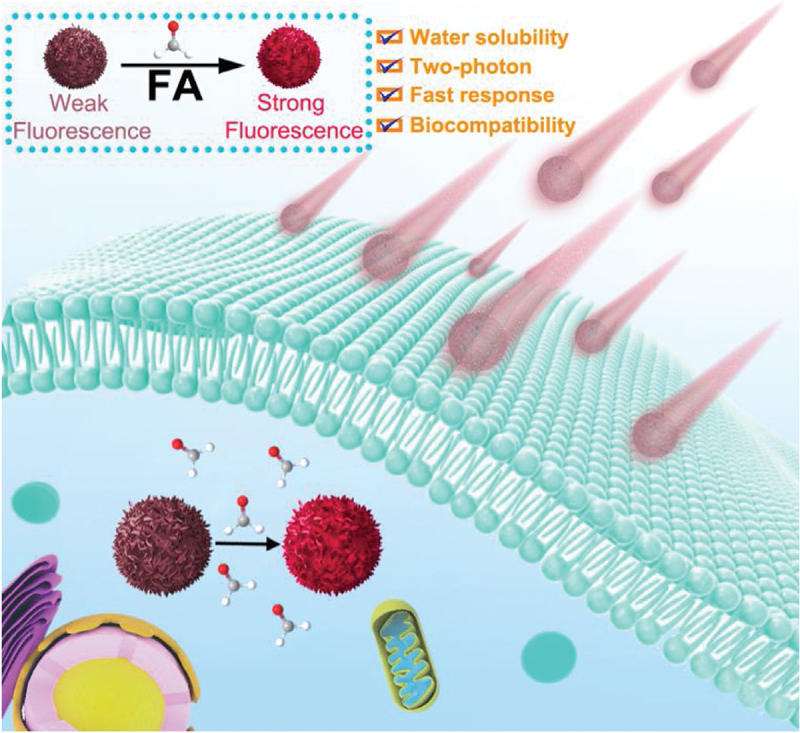
Schematic representation of the sensing via RCD for FA. Reprinted with permission from ref. [71]. Copyright (2020), Elsevier.

Red carbon dots (RCD), label-free two-photon fluorescent nanoprobes, were used for the detection and imaging of FA in live cells and zebrafishes [71]. The RCD nanoprobes exhibited a rapid and specific reaction with aldehydes through a Schiff base formation, due to the presence of -NH_2_ groups in their structure. A blue-shift emission was observed for disrupting the hydrogen bond interaction between RCD and water. Notably, these nanoprobes demonstrated impressive photostability, fast response times (under 1 min), and high sensitivity (~9.9 µmol/L). RCD was successfully introduced to monitor FA in live cells and zebrafishes using one-photon microscopy (OPM) and two-photon microscopy (TPM) for its excellent water dispersity and biocompatibility (Figure 6).

A novel self-assembled fluorescent nanoparticle, CS-OCH_3_@NBHN, was developed for the rapid detection of FA [72]. This nanoparticle was crafted using a chitosan-based fluorescent probe (CS-OCH_3_) as a carrier, loaded with a small-molecule FA fluorescent probe. The CS-OCH_3_@NBHN nanoprobe exhibited remarkable speed and sensitivity in recognizing FA, with a ratiometric fluorescence response occurring in less than 10 min. It functioned effectively within a pH range of 3.0–11.0, demonstrating high selectivity, minimal interference, and a low detection limit of 66.3 nM. Furthermore, the CS-OCH_3_@NBHN probe successfully identified FA in various samples including aqueous solutions, air, leather, and cells, indicating its promising application prospects.

## 4. Metal-organic framework (MOF)-based FA sensors

Metal-organic framework (MOF) is a new class of functional porous materials having extended crystalline frameworks, comprising metal ions and organic linkers [73]. Owing to the tuneable functionality and porosity, chemical stability as well as highly conjugated backbones, MOF materials have been widely employed in chemical sensing [74]. Apart from MOFs, covalent organic polymers (COPs) are another class of crystalline porous polymeric materials with a pre-designable porous structure [75], diverse functionalities, and high thermal stability [76]. The systematic pore surface engineering of COPs can favourably offer tailor-made covalent docking for diverse functional groups like aminals [77], amines [78], and imines [79] with controlled loading contents to the pore walls. Using MOFs, Zhao et al. first demonstrated a zeolitic imidazolate framework (ZIF-8) material for indoor FA sensing [80]. Nandi et al. fabricated a hydrazine functionalized Al(III)-based MOFs for the sensitive and selective detection of FA [81]. The fluorescence ‘turn-on’ behaviour of the reaction-based probe can be ascribed to the inhibition of the PET process (from the hydrazine group to the phenyl ring) because of the formation of the hydrazone moiety. The detection limit of the probe was reported as 8.37 μM (0.25 ppm) with a short response time (1 min) for FA in 10 mm HEPES buffer. Vellangiri and coworkers selectively detected FA by using four materials, two water-stable MOFs of UiO-66 (U6) and U6-NH_2_ (U6N), and two COPs with amine-functionality, CBAP-1-EDA (CE) and CBAP-1-DETA (CD). U6N exhibited the highest removal capacity, which was 2 times higher than that of the reference sorbent, activated carbon [82].

**Figure 7.**
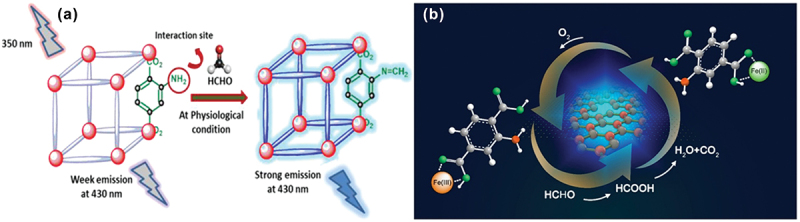
(a) “Turn-On” sensing of FA by the d10-MOF (CMERI-1) under physiological condition. Reprinted with permission from ref. [83]. Copyright (2021), American Chemical Society. (b) Schematic representation of ZIF-8-based MOFs for FA sensing. Reprinted with permission from ref. [85]. Copyright (2021), Elsevier.

Recently, two luminescent porous networks (CMERI-1 and CMERI-2) were reported for the efficient detection of FA in an aqueous medium (Figure 7a) [83]. The framework CMERI-1 showed better sensitivity with a very short response time (1 min) in the realm of FA detection due to the facile imine (−N=CH−) formation, which was restricted in the case of CMERI-2. The fluorescence ‘turn-on’ behaviour was ascribed to the inhibition of the PET process (from the amine subunit to the secondary building unit). The detection limits of CMERI-1 and CMERI-2 toward FA in aqueous medium were found to be 0.62 μM (0.019 ppm) and 1.39 μM (0.041 ppm), respectively. In addition to the solution-phase studies, a MOF-based hydrogel membrane was also fabricated, which showed vapor-phase detection of FA. Fang *et al.* reported 4,4’-(1,2-diphenylethene-1,2-diyl)dibenzoic acid-based supramolecular organic frameworks (HNU-44) for the detection of FA within 20 s [84]. The gaseous FA in contact with HNU-44 formed the hydrogen bond between the FA and (Z)-4,4′-(1,2-diphenylethene-1,2-diyl)dibenzoic acid (H_2_DCPE), which activated the intramolecular rotation and caused fluorescence quenching. The HNU-44 showed LOD values of 27.41 ppb and 2.61 ppb for the solution and gas phases, respectively. Furthermore, the FA content in an actual fish sample was also detected by HNU-44. Yan et al. reported NH_2_-Fe(III)-nMOFs with high PL, porosity, and strong oxidizing ability [85]. The FA sensing platform was developed based on the specific interaction between active metal sites of NH_2_-Fe(III)-nMOFs and intermolecular hydrogen bonding between FA and NH_2_-Fe (III)-nMOFs. Importantly, the prepared NH_2_-Fe(III)-nMOFs captured FA through both active metal sites of NH_2_-Fe(III)-nMOFs and hydrogen bonding. Meanwhile, the captured FA was oxidized by active metal sites of NH_2_-Fe(III)-nMOFs and converted to methanoic acid (MA) and then transformed to CO_2_ and H_2_O *via* the oxidation-reduction cycle of NH_2_-Fe(III)-nMOFs-FA-O_2_ (Figure 7b).

## 5. Polymeric/Hybrid materials-based probe

Polymeric sensors offer several advantages over other materials due to their structural flexibility [86], tuneable molecular weight, solubility in a wide range of solvents, low-cost production, etc. [87,88]. Interestingly, polymeric sensors show the cooperative effect of many recognition sites in a single polymer chain, thereby increasing the binding efficacy towards analytes [89]. Moreover, hydrophobic sensing moiety can be integrated into water-soluble functional polymers *via* post-polymerization modification or copolymerization methodologies. In the following section, recent advancements in polymeric probes for FA sensing are discussed in both solution and vapour phases.

A quartz crystal microbalance (QCM)-based FA sensor was prepared by coating electrospun polyacrylonitrile (PAN) nanofibrous membranes with polyvinyl amine (PVAm) onto the electrode surface. The PAN nanofibrous membranes provided various benefits, such as large surface area, high porosity, and stiffness. The PVAm with a huge number of primary amine groups interacted strongly with FA [90]. The hierarchical structure of PAN nanofiber helped to improve the sensing capabilities. The constructed QCM sensors showed low detection limits of 500 ppb within a short detection time of 2 min. Furthermore, the reversible nucleophilic addition reaction process between FA and the primary amine groups in PVAm resulted in the QCM sensors with features such as reversibility, great repeatability, and excellent selectivity.

**Figure 8.**
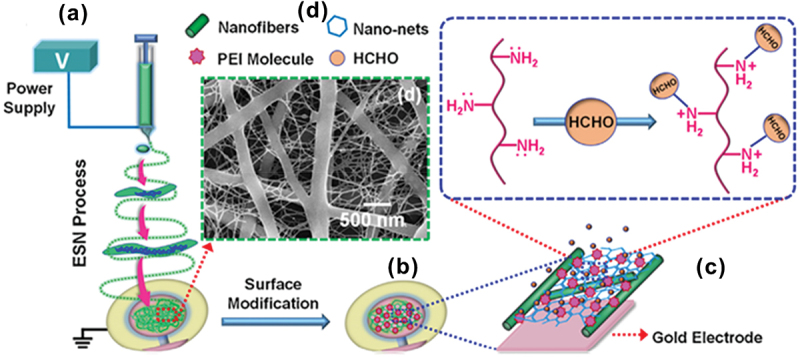
(a) Electrospun nanofibrous chitosan (ESN) deposition of fibrous membranes onto the QCM electrode. (b) Surface modification of NNB with PEI solutions. (c) Reaction mechanism between FA and PEI. (d) Field emission scanning electron microscopy (FESEM) image of NNB structured chitosan-based membrane. Reprinted with permission from ref. [91]. Copyright (2014), Elsevier.

A polyethyleneimine (PEI) functionalized chitosan with QCM nanofiber binary layer was developed for FA sensing (Figure 8a) [91]. To obtain the well-defined uniformly distributed structure of the PEI, nanofibers and spider-web-like nanonets were constructed using a simple electro-spinning procedure (Figure 8b). The multi-dimensional nanostructure fabrication was possible using QCM for FA gas sensing analysis (Figure 8c). The nucleophilic addition elimination reaction between FA molecules and primary amine groups of PEI leads to the increase in FA absorption into the surface of the membrane. QCM sensor demonstrated trace amount of FA detection from the environment with a detection limit of 5 ppm. Chitosan/SDS (0.1 wt%) solutions created the best nanofibers-net-binary (NNB) structured membranes, with a high porosity and specific surface area of 8.25 g/m^2^, enlarging the sensing area and facilitating the passage of FA into the PEI-modified sensing layers (Figure 8d).

To keep track of the trace-level concentration of FA gas at room temperature, Tai and coworkers developed a QCM-based gas sensor [92]. Since FA gas can be chemically adsorbed by polymeric amines *via* a reaction of primary amines to form azomethine or Schiff base, mixed PEI-multiwalled carbon nanotubes (MWCNTs) were sprayed on QCM. The volume ratio of PEI to MWCNTs of 1:1 was proven to be most effective with superior sensing properties. The optimized sensor showed a linear fit of the frequency shift *versus* FA concentration within 6 ppm. The sensor had a significantly lower LOD of 0.6 ppm. The sensor was examined by exposure to other various gases, and it was only selective to FA gas with a sensing capacity of within 6 ppm, with good reproducibility. Although the sensor lacked a long-term stable response/recovery rate, this simple spraying fabricated QCM system proved worthwhile for sensitive and fast detection of FA at room temperature.

**Figure 9.**
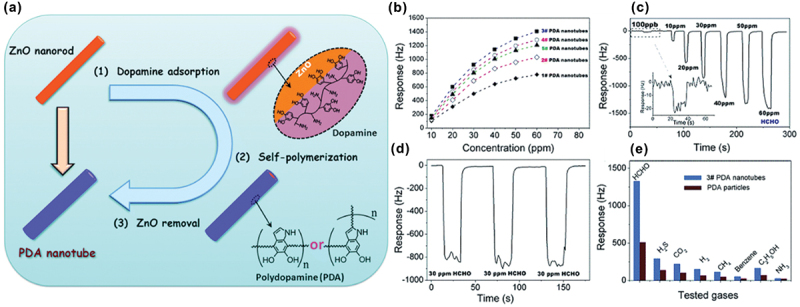
(a) Synthesis scheme of the PDA nanotubes. (b) Responses of the different FA concentrations in the range of 10-60 ppm. (c) Real-time dynamic response curve with increasing FA concentration at room temperature. (d) Repeatable and reversible sensing response of the PDA nanotube sensor to 30 ppm of FA. (e) Responses of PDA nanotubes and amorphous PDA with various interference analytes. Reprinted with permission from ref. [93]. Copyright (2016), Royal Society of Chemistry.

Yan et al. reported polydopamine (PDA) nanotubes, synthesized by using sacrificial templates of ZnO nanorods [93]. The dopamine molecules were self-polymerized in an alkaline solution to form the PDA nanotubes (Figure 9a). The thickness of the nanotube walls varied from 13 to 75 nm by controlling the polymerization time. The dopamine molecules, possessing both catechol and amine groups, were self-assembled onto the surface of ZnO nanorods, as mussels strongly adhere to inorganic or organic substrates. The PDA nanotubes were uniformly deposited over a QCM to create a gas sensor. The PDA nanotubes showed remarkable FA detection abilities due to their large surface area and a huge number of amine groups (Figures 9b, c). The enthalpy value (Δ*H°* = −53.6 kJ/mol) during the adsorption process of FA onto PDA nanotubes was determined through temperature-varying microgravimetric measurements. This value was further confirmed using theoretical calculation. Thermodynamic experiments indicated the suitability of PDA nanotubes for FA detection due to their moderate -Δ*H°* value. The sensor exhibited a detection limit of less than 100 ppb for FA (Figures 9d, e).

Since PDA chemically adsorbs the gas molecules due to the presence of catechol, amine, and amine functional groups, Song et al. prepared a QCM sensor for selective and sensitive FA gas detection, based on SnO_2_ nanofibers (NFs)/PDA nanocomposite [94]. A composite film with a volume ratio of SnO_2_ NFs to PDA = 1:2 was seen to be most efficient for FA detection and thus selected as the QCM sensing layer. It can detect over a wide range of FA concentrations (0.5–50 ppm), with an LOD as low as 500 ppb, ensuring its high sensitivity (12.2 hz/ppm). This could be allocated to hydrogen bonding and a combination of aldehyde amine Schiff base interactions. This sensor had a very short response and recovery time of 25 s and 38 s, respectively. Various other gases were exposed to the sensor, but the affinity of the sensor towards FA gas was significantly higher than that of other gases, indicating its high selectivity.

Feng and coworkers developed an amine-terminated polymeric probe for toxic FA detection [95]. They used regular pH indicators in a polymer film doped into a poly(ethylene glycol) (PEG) polymer, which detected FA concentrations of 250 ppb to 20 ppm in less than 1 min and almost 50 ppb of FA within 10 min. Upon interaction of the amine groups present in the polymeric probe with FA, an alteration of pH occurred, causing the indicators to exhibit a colorimetric change. A fully functional prototype device was constructed and tested, utilizing an inexpensive white light-emitting diode (LED) and an ordinary complementary metal oxide semiconductor (CMOS) camera. Combined with a low-dead-volume cartridge, this handheld device provided a rapid, highly sensitive, and quantitative method for the portable monitoring of FA at various concentrations.

**Figure 10.**
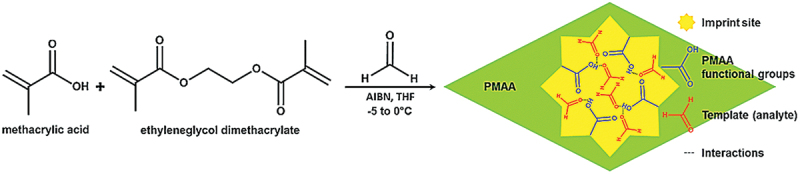
Synthesis scheme of imprinted poly(methacrylic acid) (imp-PMAA). Reprinted with permission from ref. [96]. Copyright (2014), Royal Society of Chemistry.

Iqbal et al. reported an imprinted poly (methacrylic acid) (imp-PMAA) and gold nanoparticles (PMAA/Au-NPs) composite for sub-ppm level detection of indoor air pollutants [96]. The polymer matrix was prepared *via* free radical polymerization under UV light at 0°C, where methacrylic acid (MAA) monomer, ethylene glycol dimethacrylate (EGDMA) crosslinker, and azobisisobutyronitrile (AIBN) initiator were reacted in tetrahydrofuran (THF) in the presence of FA vapour as imprinting material (Figure 10). Layer-by-layer assembly was used to fabricate the device. Au-NPs were synthesized by mixing tetrakis(hydroxymethyl)phosphonium chloride (THPC, 80% aqueous solution) and aqueous HAuCl_4_ (0.1 M) in NaOH solution. The Au-NPs water suspension showed absorption at 520 nm. PMAA was chosen for FA imprinting due to non-covalent interactions with carboxylic acid groups and enhanced sensor performance. This sensor exhibited 2 to 9-fold increase in response towards gaseous FA (1 ppm) compared to other tested materials. It was susceptible to gaseous FA with low cross-sensitivity toward interfering gases and humidity. It showed a low LOD of 152 ppb, fast response and recovery time, complete reversibility, good short-term repeatability, and minimal humidity effect.

**Figure 11.**
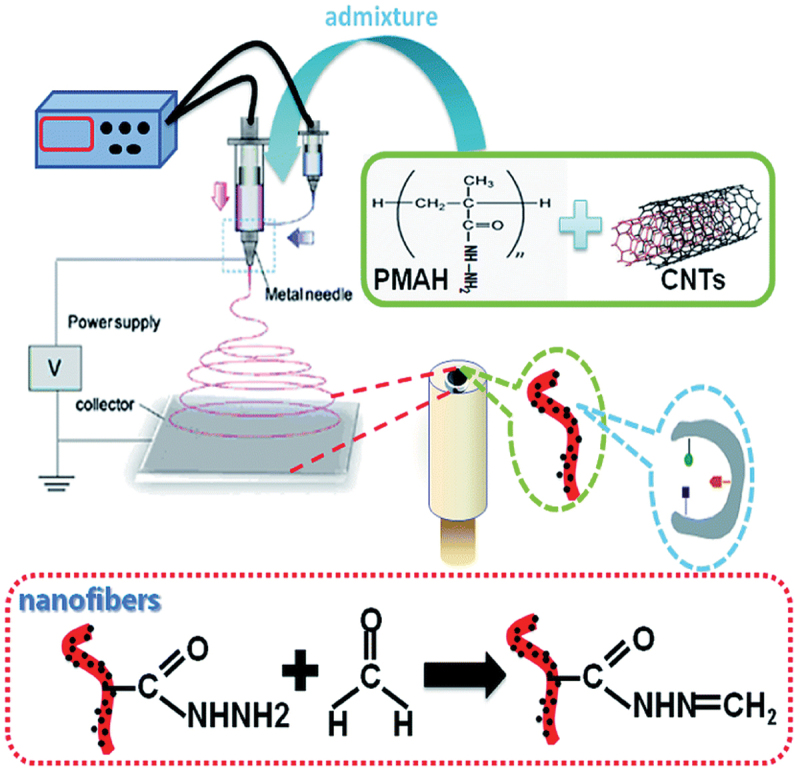
Schematic representation of the biomimetic sensing platform for FA. Reprinted with permission from ref. [97]. Copyright (2015), Royal Society of Chemistry.

Electrospun biomimetic recognition nanofibers provide several advantages, like sensitivity, stability, and selectivity in detection. Thus, electrospun nanofibers were introduced for FA detection [97]. The electrospun nanofibers were employed as the bio-recognition element to fabricate biomimetic sensors, based on electrochemical impedance spectroscopy (EIS). The combination of electrospinning and molecular imprinting hybrid technique was utilized, offering advantages over traditional biomimetic materials (Figure 11). To enhance the conductivity of the electrospun polymer nanofibers, carbon nanotubes were incorporated into the nanofiber structure. This functionalization allowed for the fabrication of a biomimetic sensor platform, where the recognition sites on the nanocomposite fibers exhibited noticeable changes in response to FA molecules.

An amperometric nano-bioelectrode with *π-π* stacked 1-pyrenebutyric acid (PBA) units and carboxylated multiwalled carbon nanotubes (MWNTs) on the electrode surface allowed covalent attachment of *β*-nicotinamide adenine dinucleotide sodium salt (NAD^+^) dependent FA dehydrogenase (FDH) [98]. The FDH bioelectrode was characterized by Fourier transform infrared spectroscopy (FT-IR), Raman spectroscopy, and EIS. The resultant enzyme bioelectrode showed a large dynamic range for FA detection from 10 ppb to 10 ppm. In comparison to the stirred solution approach, the flow injection analysis showed a lower detection limit and increased affinity for FA (apparent Michaelis–Menten constant (*K*_M_) = 19.9 ± 4.6 ppm). The bioelectrode was selective towards FA, although it showed a small amount of cross-reactivity for acetaldehyde (25%) and none for propanaldehyde, acetone, methanol, and ethanol. This strategy offered a potential nano bioelectrode design for the clinically relevant ultralow-level non-invasive detection of small molecule markers in cancer and other disorders, even in complicated matrices.

**Figure 12.**
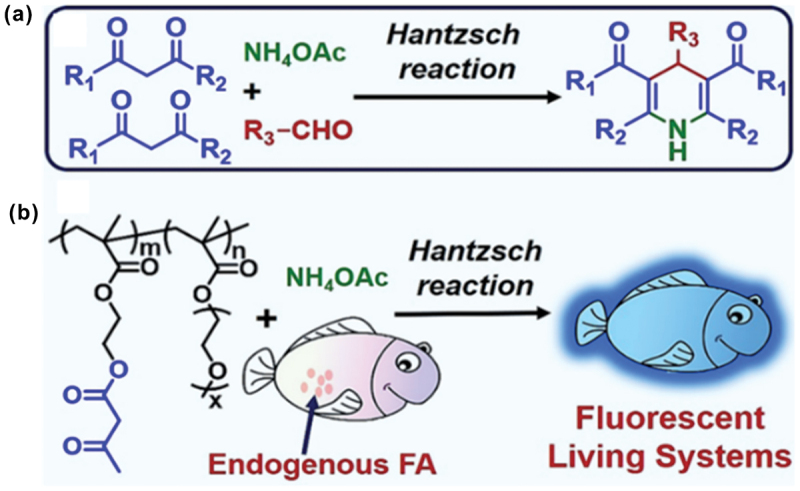
(a) Hantzsch reaction for small molecule. (b) Copolymer for the detection of FA via the Hantzsch Reaction. Reprinted with permission from ref. [99]. Copyright (2018), American Chemical Society.

Liu and coworkers successfully developed a polymeric probe, which enabled the detection of endogenous FA in living systems using the Hantzsch reaction (Figure 12) [99]. The synthesized polymers not only served as useful probes for bioimaging purposes but also expanded the application potential of the Hantzsch reaction. The polymer solution displayed high fluorescence after only 5 min of incubation with FA, confirming their potential as probes for detecting FA. The advantage of this approach was that it allowed the straightforward preparation of large amounts of polymers, avoiding laborious multistep syntheses required for other types of probes.

**Figure 13.**
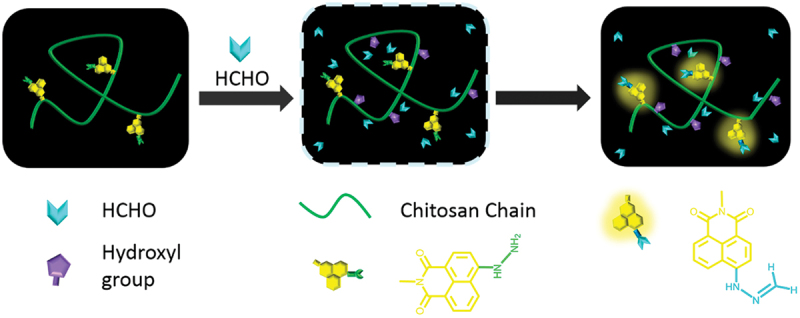
The sensing mechanism of the HN-Chitosan-based polymeric probe. Reprinted with permission from ref. [100]. Copyright (2018), American Chemical Society.

The detection of FA in aqueous solutions is crucial due to its presence in aquatic food from illicit addition or improper storage [100]. While small molecule-based fluorescent probes offer selectivity, they face challenges like slow response time and limited sensitivity. Addressing these, a novel polymeric probe was introduced, consisting of hydrophilic hydrazino-naphthalimide-functionalized chitosan (HN-Chitosan). This probe exploited specific chemical reactions between FA and grafted hydrazino-naphthalimide groups, leading to a rapid fluorescence response. Unlike small molecule counterparts, HN-Chitosan utilized cooperative binding effects, enhancing sensitivity and response time. Its ultrafast equilibrium fluorescence response (under 1 min) and high sensitivity enabled effective detection in real-world food and water samples. The modular design principle suggested broader applicability in constructing polymeric probes for swift detection of various pollutants, promising advancements in environmental monitoring and safety assurance (Figure 13).

**Figure 14.**
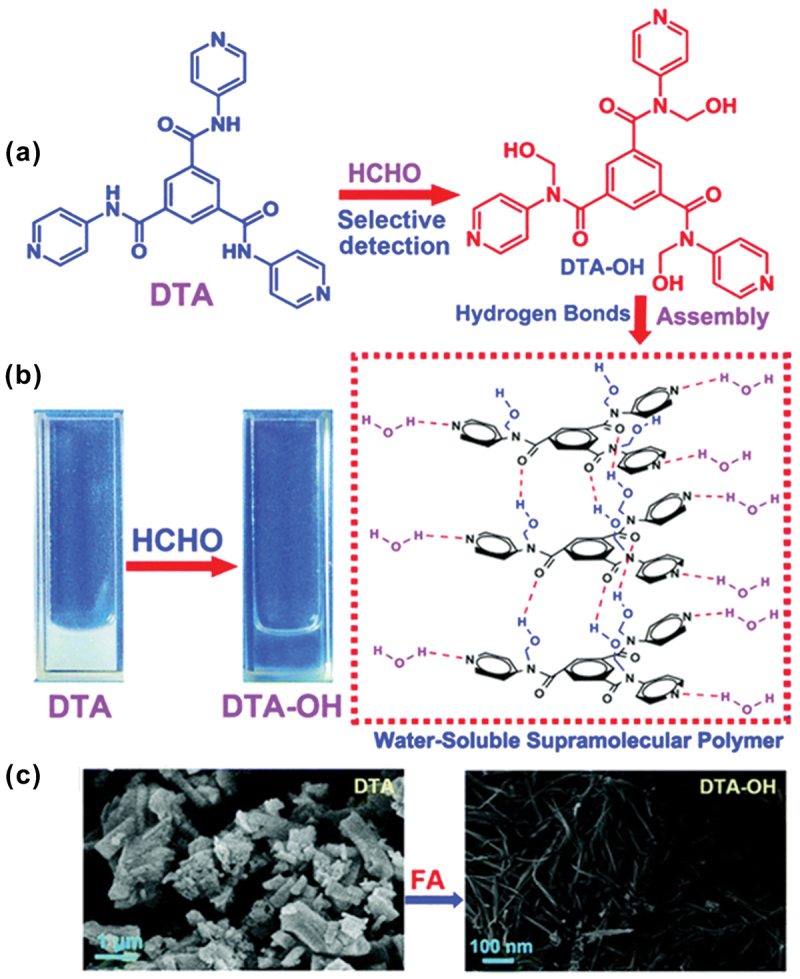
(a) Proposed reaction mechanism of DTA-based FA sensor. (b) Photograph of DTA before and after the addition of FA. (c) Morphological change (SEM image) of DTA before and after FA addition. Reprinted with permission from ref. [101]. Copyright (2019), Royal Society of Chemistry.

Fan et al. reported a probe based on a host compound N^1^, N^3^, N^5^-tri(pyridin-4-yl)benzene-1,3,5-tricarboxamide (DTA) [101]. DTA can adsorb FA efficiently and detect FA from its water solution by forming a water-soluble supramolecular polymer. Hydroxymethylation interaction occurred between the imino group of DTA and FA to form a carbinolamine compound (DTA-OH) (Figure 14a), which assembled into a water-soluble supramolecular polymer (Figure 14b). The probe displayed selectively towards FA over other aldehydes. The interaction of DTA with FA resulted in a change in solution color (under UV light, a light blue fluorescence *λ*_em_ = 470 nm was observed in Figure 14b) and a supramolecular self-assembled structure (SEM study confirmed fiber structure in Figure 14c). The detection limit for FA was found to be 1.79 × 10^−8^ M, and in the detection process, DTA acted as an adsorbing material.

**Figure 15.**
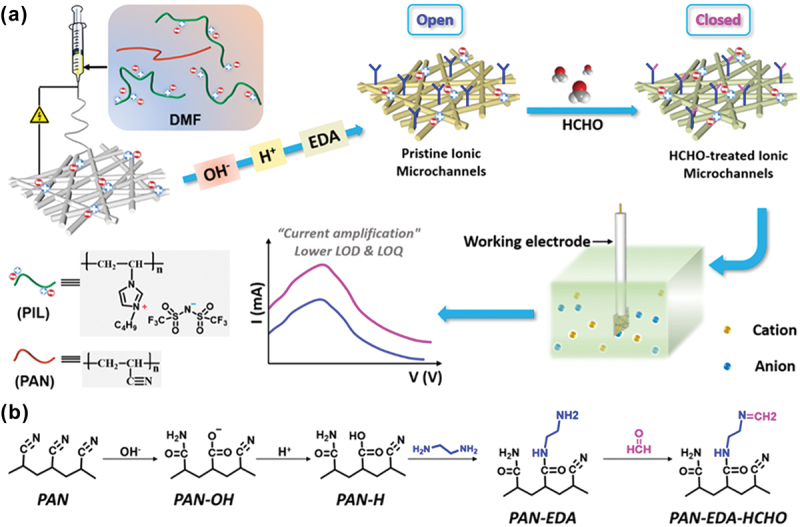
(a) Schematic roadmap of ultrasensitive recognition of FA, by using poly(ionic liquid)/polyacrylonitrile nanofibrous membrane (PIL/PAN NFM). (b) The reaction mechanism of the designed ionic microchannels with FA. Reprinted with permission from ref. [102]. Copyright (2020), American Chemical Society.

An ionic microchannel for FA detection was reported, where ethylenediamine (EDA)-functionalized poly(ionic liquid)/polyacrylonitrile nanofibrous membrane (PIL/PAN NFM) was fabricated (Figure 15a) [102]. The reactivity of FA with the EDA-immobilized nanofibrous membrane allowed the switch of ionic current output from low to high. This was attributed to the decrease in zeta potential and increase in electron affinity of the microchannels upon interaction with more FA. The amplified ionic current signals due to the formation of more ion transport paths along the ionic nanofibers enabled the fabrication of microchannels for the detection of trace amounts of FA in an aqueous solution (Figure 15b). By exploiting this ionic current amplification mechanism, the sensitivity and accuracy of the detection of FA can be improved significantly. Furthermore, the combination of nanofibers and microchannels provided a highly efficient and economical solution for detecting FA in an aqueous solution.

Mavani and Penlidis prepared polyaniline (PANI) and its derivative, poly(2,5-dimethyl aniline), and studied their sensitivity towards FA. Polyaniline demonstrated higher sensitivity compared to its derivative [103]. To achieve better sensitivity, PANI was doped with different weight percentages of indium oxide (In_2_O_3_) and subsequently evaluated for sensing capabilities, including sensitivity, selectivity, and stability. The PANI with 1.25 wt% of In_2_O_3_ exhibited the highest sensitivity to FA, whereas PANI with 5 wt% of In_2_O_3_ displayed the greatest selectivity for FA over benzene interference. Interestingly, the trends in sensitivity and selectivity of PANI doped with different weight percentages of In_2_O_3_ were found to be inversely correlated. PANI with 1.25% doped In_2_O_3_ showed high sorption performance due to its unique sheet-like layered surface morphology.

**Figure 16.**
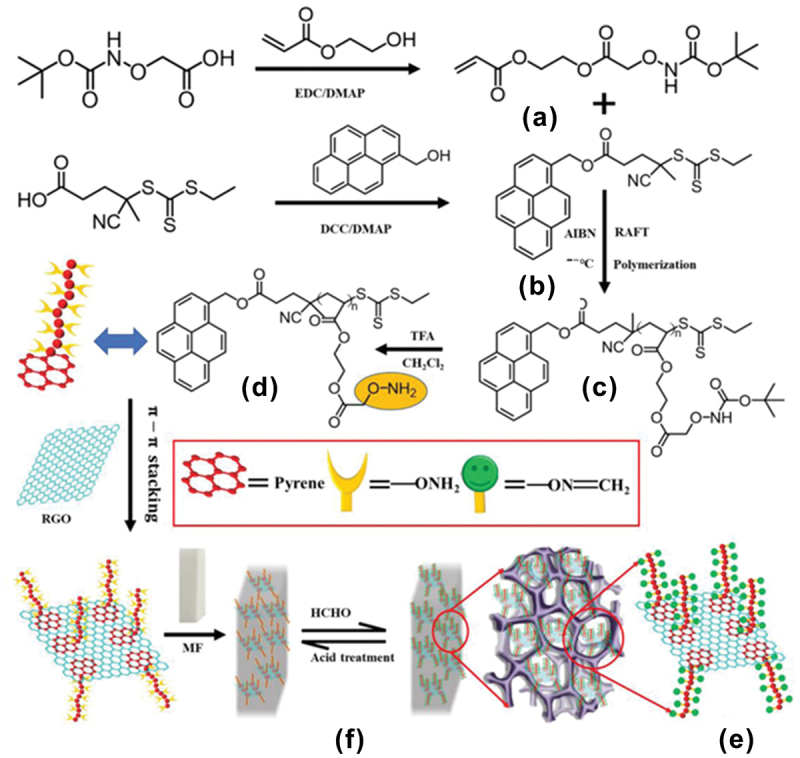
(a) Synthesis scheme of monomer, (b) pyrene functionalized RAFT agent, (c) protected polymer, (d) deprotected polymer, (e) fabrication of polymer/RGO composite via self-assembly and π–π stacking, and (f) loading into melamine foam for chemical removal of FA. Reprinted with permission from ref. [104]. Copyright (2022), WILEY-VCH.

Reversible addition-fragmentation chain transfer (RAFT) polymerization was used to synthesize -ONH_2_-functionalized poly(2-hydroxyethyl acrylate) (pHEA-ONH_2_) with controlled molecular weight, narrow polydispersity index (PDI), and terminal -ONH_2_ groups [104]. The polymer (pHEA-ONH_2_) easily immobilized on reduced graphene oxide (RGO) *via* strong *π-π* stacking to afford a stable RGO/pHEA-ONH_2_ composite which was loaded into the 3D skeleton of melamine foam (MF) to afford RGO/pHEA-ONH_2_/MF for efficient removal of gaseous FA (Figure 16). The chemical removal of FA was achieved by a nucleophilic addition reaction between FA and aminooxy groups on the polymer chains to form the oxime bonds with the elimination of water. Each gram of polymer could remove about 14 mg of FA at room temperature. The capability of the composite to chemically remove gaseous FA in ultralow concentration at ambient temperature with high efficiency may find promising commercial applications in air purification for apartments, offices, and other sealed environments like trains, ships, airplanes, submarines, etc.

**Figure 17.**
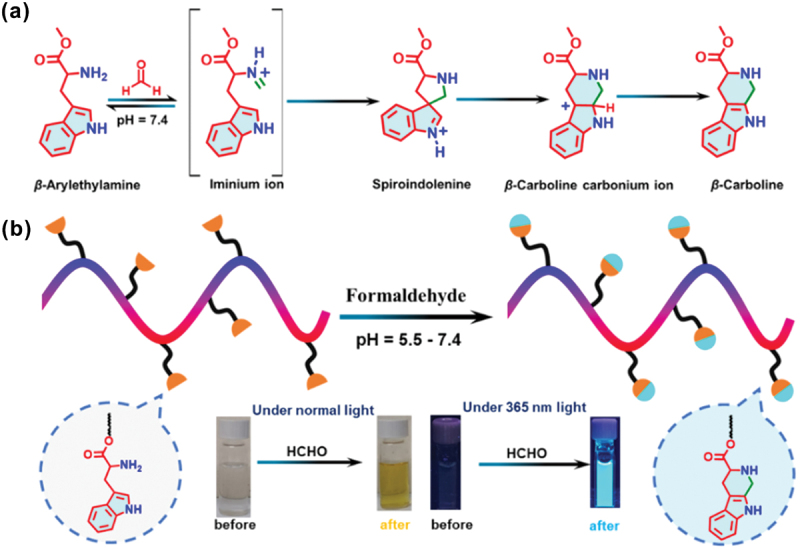
(a) Pictet-Spengler reaction mechanism with the small molecule analog of the BTME monomer. (b) Schematic illustration of the FA sensing. Reprinted with permission from ref. [105]. Copyright (2014), Elsevier.

We recently reported a water-soluble polymeric probe with tryptophan pendants for FA sensing in an aqueous medium [105]. The RAFT polymerization technique was employed to synthesize the copolymers of *N,N*-dimethylacrylamide (DMA) and *tert*-butyl carbamate (Boc)-tryptophan methacryloyloxyethyl ester (BTME). To obtain the desired polymeric probe with pendant primary amine groups, Boc groups were deprotected from the tryptophan moieties. Tryptophan was strategically introduced as a signal-reporting unit in the polymeric probe due to its remarkable structural flexibility, photostability, and availability. The sensing mechanism was based on the FA-induced selective Pictet-Spengler cyclization reaction (Figure 17a). The polymeric probe exhibited cyan fluorescence in an aqueous medium after interacting with FA due to the formation of a *β*-carboline derivative. The copolymer was found to be sensitive to nanomolar levels of FA in aqueous solutions, with a very fast reaction time of 2 min, due to the cooperative binding impact of the many recognition sites and nearby amine groups. The probe selectively detects FA by colorimetric and fluorometric methods, with a detection limit as low as 25 nM (Figure 17b). In this section, we discussed different types of polymers for selective and sensitive detection of FA *via* colorimetric, fluorometric, electrochemical, morphological, and micro-gravimetric methods. Various types of polymeric probes, detection limit, LOD, and their applications are summarized in Table 2.

**Table 2.** Various fa-sensing polymeric probes with their application details.

Polymeric Probe	Detection phase	Detection limit	Detection time	Application	Ref.
Polyvinyl amine	gas	500 ppb	2 min	-	90
PEI functionalized chitosan	gas	5 ppm		-	91
PEI-MWCNTs based	gas	0.6 ppm	-	-	92
PDA nanotubes	gas	-	-	-	93
QCM based	gas	500 ppb	-	-	94
Amine functinalized PEG	gas	250 ppb	1 min	-	95
Imp-PMAA/Au-NPs hybrid sensor	gas	152 ppb	-	-	96
Electrospun nanofibers	aquous	-	-	-	97
Amperometric nano-bioelectrode	aquous	10 ppb − 10 ppm	-	-	98
Hantzsch reaction based	aquous	3.1 × 10^−7^ and 3.4 × 10^−7^ M	5 min	Living cells	99
Naphthalimide functionalized chitosan	aquous	1.66 µM	<1 min	Sensing in tap water, chicken, etc.	100
DTA-based probe	aquous	1.79 × 10^−8^ M	-	-	101
EDA-functionalized poly (ionic liquid)/polyacrylonitrile	aquous	0.036 ppt	-	Home water monitoring	102
PANI and derivates	gas	-	-	-	103
Poly (2-hydroxyethyl acrylate)	gas	-	-	Air purification	104
Tryptophan based	aquous	25 nM	2 min	-	105

In the above discussion, QCM-based sensors with specific chemical coatings, such as polymers, selectively detected mainly gaseous FA. Table 2 provides an overview of various types of polymeric/hybrid probes, along with the detection phases (gas or solution), and their specific applications in FA sensing.

## 6. Conclusion and future perspective

**Figure 18.**
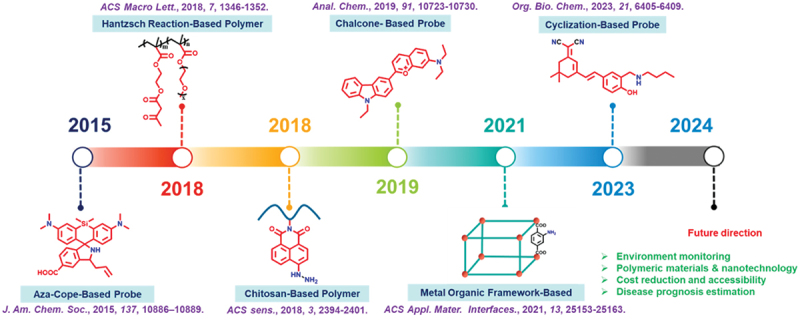
Roadmap journey of different types of FA sensors.

This review article outlines the pioneering evolution of various probes designed for sensitive and selective detection of FA, with a primary focus on the benefits offered by polymeric probes. Figure 18 shows the evolution of FA-based fluorescent probes with a chronological progression through different stages. Different types of fluorescent sensors have been engineered using reaction mechanisms such as the aza-Cope rearrangement, FA-amine condensation reaction, etc., enabling remarkable selective sensing of FA. In the mechanism developmental stage, researchers established many fluorescent probes to improve the spectroscopic properties, sensitivity, selectivity, and cytotoxicity of the probes [106]. The exploration stage focuses on improving the bio-application of the probes, such as through NIR, reversible, analyte regeneration, and gene encoding probes. Various small molecular probes were designed as effective fluorescent imaging agents to detect both exogenous and endogenous FA within different living cells. Beyond the realm of small molecular probes, researchers have delved into the potential and advantages offered by polymeric probes, surmounting challenges like low aqueous solubility, high detection limits, and extended exposure times commonly encountered with small molecular probes and other materials. Growing evidence supports that the reaction-based colorimetric method stands out as a prominent platform for FA detection, as it triggers a visible colour change following the selective addition of FA, crucial for real-life applications. As FA continues to be a significant environmental and health concern, future research in FA sensing holds great promise for addressing emerging challenges and expanding the application domains of FA detection technologies.

The development of advanced materials for FA detection is primarily focused on creating probes that deliver fast, convenient, cost-effective, and accurate measurements with minimal detection limits. In biological contexts, there is an increasing demand for probes that can penetrate deeper tissues, ensure precise targeting, and offer enhanced spatiotemporal resolution. Probes with reversible or FA-regenerated properties are particularly advantageous, as they can maintain local FA concentration and homeostasis during detection [107]. Additionally, the development of multifunctional probes that can detect multiple analytes simultaneously is vital for exploring complex physiological processes. These multifunctional probes can provide comprehensive insights into various biological systems by simultaneously tracking multiple parameters. Integrating multimodal imaging techniques, such as combining fluorescence imaging with magnetic resonance imaging (MRI), photoacoustic imaging (PAI) [108], and positron emission tomography (PET)-holds great promise for improving the translatability and applicability of these probes. Such multimodal approaches enable thorough monitoring and analysis, facilitating a more holistic understanding of biological systems. These advancements in FA detection materials have broad applications, ranging from clinical diagnostics to fundamental research in biochemistry and molecular biology. The pursuit of these advanced tools aims to address the growing need for sophisticated technologies in biomedical imaging and molecular sensing, ultimately contributing to enhanced health outcomes and more effective medical treatments [109]. The future of polymeric materials in formaldehyde sensing is promising due to their versatility, cost-effectiveness, and potential for integration with modern technologies [110]. Continued research and development in this field are expected to yield highly efficient, reliable, and user-friendly sensing solutions.
